# Biomechanics of esophageal elongation with traction sutures on experimental animal model

**DOI:** 10.1038/s41598-022-07348-4

**Published:** 2022-03-01

**Authors:** Krystian Toczewski, Sylwester Gerus, Maciej Kaczorowski, Marta Kozuń, Justyna Wolicka, Kamila Bobrek, Jarosław Filipiak, Dariusz Patkowski

**Affiliations:** 1 Department of Paediatric Surgery and Urology, Wrocław Medical University, Borowska 213, 50-556 Wrocław, Poland; 2 Department of Clinical and Experimental Pathology, Wrocław Medical University, Borowska 213, 50-556 Wrocław, Poland; 3 Department of Mechanics, Materials and Biomedical Engineering Mechanical Faculty, Wrocław University of Science and Technology, Łukasiewicza 7/9, 50-371 Wrocław, Poland; 4 Department of Epizootiology and Clinic of Birds and Exotic Animals, Faculty of Veterinary Medicine, Wrocław University of Environmental and Life Sciences, pl. Grunwaldzki 45, 50-366 Wrocław, Poland

**Keywords:** Biomaterials, Experimental models of disease, Paediatric research

## Abstract

Esophageal elongation is one of the methods of long gap esophageal atresia treatment. The aim of the study was to determine the best type of traction suture for esophageal lengthening on an animal model. White Pekin Duck’s esophagi were used as a model (fresh-frozen and thawed). The esophagus was cut in half, then both ends were sutured together and extended on a tensiometer. Tested sutures involved simple suture, suture aided by a single or double clip, and suture aided by pledget (10 samples each). Constant and 2 methods of intermittent traction were also compared. The histological study showed similarities between duck’s and newborn’s esophagus. The highest maximal force was achieved with pledget suture (F = 8.59 N ± 1.45 N), then with double clip (F = 5.74 N ± 1.29 N) and the lowest with single suture (F = 3.80 N ± 0.54 N) (*p* < 0.001). Pledget suture also allowed for the greatest elongation (*p* < 0.01). Intermittent traction results in better elongation at the same breaking strength as constant traction (*p* < 0.05) if traction is maintained during breaks. Reinforced sutures (pledget or double clip) should be taken into consideration in internal traction. When performing traction sutures, it is worth step by step carefully tightening the sliding knot in short periods before its final binding.

## Introduction

Long gap esophageal atresia (LGEA) poses a challenge to pediatric surgeons all over the world. There are different techniques in use to bridge the gap between esophageal ends based on native esophagus preservation or replacement^1^. All of these present their disadvantages and there will be no unanimity until one method is improved to outperform all the others. Patkowski’s method of internal traction is the preferred treatment for LGEA in our Department with benefits of gastrostomy avoidance and preservation of the native esophagus^2^. A serious complication of traction techniques may be the perforation of an esophageal stump. Yet no studies focused on the optimal suturing technique to achieve maximal possible elongation with the lowest risk of complications.

The aim of our study was to determine the most effective and safest suturing method for the traction. The experiment was performed on the animal model (White Pekin Duck esophagi). We compared 4 types of traction techniques and measured the differences between constant and intermittent traction.

## Materials and methods

The study was conducted on animal tissues therefore since the local law and European directive 2010/63/eu apply only to scientific experiments on living animals, no approval was needed.

### Experimental model description

Our goal was to conduct the study on a model nearly corresponding to a newborn. We chose the esophagus of the white Pekin Duck based on the availability and size of the animal which is similar to a newborn (2.5–4 kg). Esophagi (n = 60) obtained from Pekin Ducks of both sexes were delivered from the slaughterhouse directly after slaughtering, immediately dissected from remaining surrounding tissues, and deeply frozen in − 20 °C. We used the lower part of the esophagus, laying between the crop sac and the stomach, because of its wall’s elasticity as well as its round and regular shape. The specimens underwent macroscopic evaluation and all esophagi with visible damage were excluded.

To validate whether a duck esophagus constitutes an anatomically reliable model of the newborn's esophagus, representative specimens were analyzed by a surgical pathologist (MK). Selected samples were fixed in 10% buffered formalin, processed in an automated tissue processor, and embedded in paraffin. Prepared sections were stained with hematoxylin and eosin (BioOptica, Milan, Italy) and Azan stain (BioOptica) according to standard histochemical protocols.

**Figure 1:**
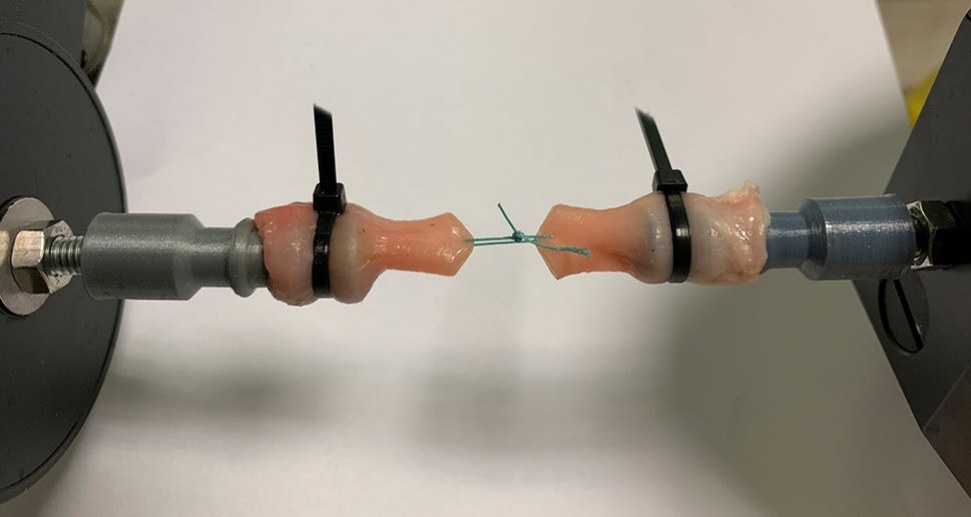
Both esophageal ends were installed on the horizontal microforce testing system and sutured with a simple suture (after prestretching).

Immediately before the experiment, the samples were thawed at room temperature. The specimens were kept moisturized with saline solution. Each sample (lower part of one esophagus) was transected into two equal parts and tied back together with a tested suture so that the distance between the puncture sites was 20 mm. Then both ends were installed on a horizontal microforce testing system (Fig. 1). We used an electronically controlled device with a resolution over 0.001 N and 0.0001 mm (Tytron 250, MTS Systems Corporation, USA). For all traction trials, force and elongation were recorded. Based on the results the force–elongation graphs were obtained and the maximal force (F_max_) and maximal elongation (DL_max_) at the point of maximal force were determined.

Tests were performed before the actual experiment to establish proper mounting and measurement technique.

### Traction sutures techniques

**Figure 2:**
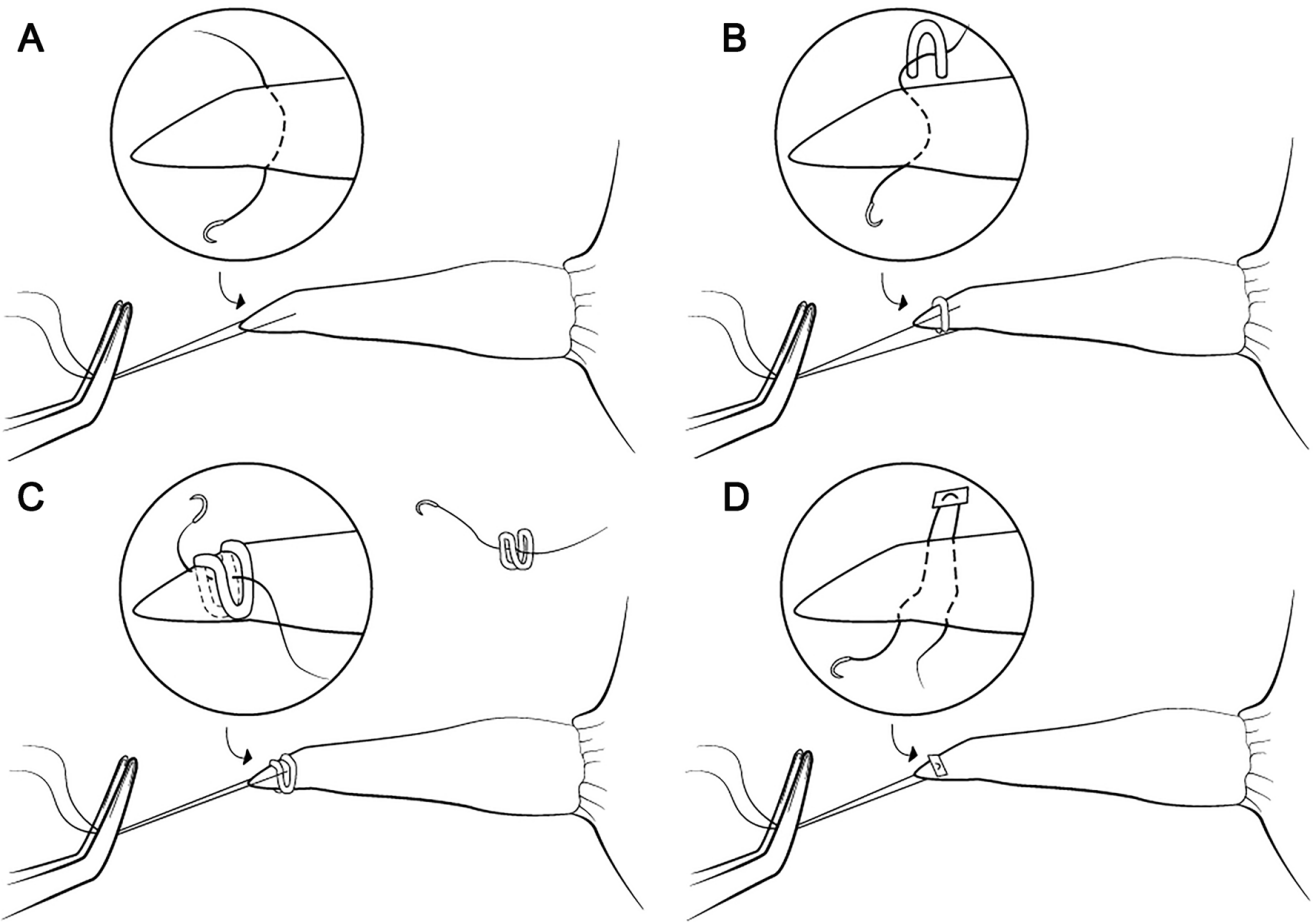
Different types of sutures: (**A**) single suture (normal binding), (**B**) single suture with a clip (single clip), (**C**) single suture with a double clip (double clip), (**D**) pledget.

A unified technique was involved for all variants by the same surgeon (KT) supervised by another author (SG). All sutures were single full-thickness and placed in the midline 3 mm from the edge of the esophagus. PreMicron 2/0 braided non-absorbable polyester sutures (BBraun) with 26 mm round needle were used. We chose 4 suture techniques based on their popularity and feasibility in internal traction in thoracoscopic long gap esophageal atresia surgery. The techniques are presented in Fig. 2 and described below.

1. Single suture (normal binding).
2. Single clip—single suture reinforced with a 5 mm titan clip (Challenger Ti-P BBraun) the clip is applied transversally after suture placement and covers full thickness of the esophagus and only one end of the thread. This suture had been prevalent for internal traction in our Department. The role of the clip is to disperse the tension to a larger tissue bite than in simple suture as well as prevent leakage from the puncture site.
3. Double clip—single suture passed through a 5 mm double clip (DS Single Fire Titanium Ligation-Clips BBraun); first, the clip is applied, then the suture is passed between the double clip’s arms. Expected benefits seem similar to single clip sutures, but the idea was to check if the double clip’s advantages surpass those of the single clip’s.
4. Pledget—pledget sutures (PreMicron 2/0 with PTFE pledgets, 26 mm round needle) which are widely used for tension suturing.

### Traction

The esophageal ends were carefully uniaxially prestretched to 1 N, which was then set as tare value. The actual traction then proceeded with a speed of 10 mm/min. The endpoint of a test was a complete rupture of the muscular layer which related to the peak force of the sample and maximal elongation of the tissue.

1. Which suture is best for internal traction?
  To check what is best for internal traction four abovementioned types of sutures were compared. For each suture type tests on 10 samples were performed.
2. Constant versus intermittent traction—is it worth waiting?

Then it was examined how the method of traction influences the forces and elongation. This was to assess the best way of initial tightening of the traction sutures during their placement at the first operation. For this experiment simple suture with a single clip was used. Two procedures of intermittent tractions were compared with constant traction.

In the first group, the traction was controlled with elongation (Elongation Control group). After typical prestretching, the esophageal ends were stretched to 1 N with an advancement of 10 mm/min and then kept in the same position for 1 min. Next, the traction was increased for another 1 N and the procedure was repeated until rupture of the esophageal wall.

In the second group, the force was used to control the traction (Force Control group). After prestretching, the esophageal ends were stretched to 1 N with an advancement of 10 mm/min, and the tension of 1 N was maintained for 1 min (the traction slowly proceeded to keep the tension). Then the traction was increased for another 1 N, and the procedure was repeated until rupture of the esophageal wall.

### Statistical analysis

The statistical analysis was conducted with IBM SPSS Statistics 25. The assumption of normal distribution for parametric tests was verified with Shapiro–Wilk and Levene’s tests. The cardinality of data was also checked. Due to meeting the assumptions, parametric tests such as single-factor analysis of variance (ANOVA) and Student’s t-test were used for independent groups. For effect size, we used η2 for the ANOVA test and Cohen’s d with the Student’s t-test. For post-hoc comparison, Tukey’s HSD test was performed. The global significance level was assumed at α = 0.05.

## Results

### Morphological analysis of the model esophagus

**Figure 3:**
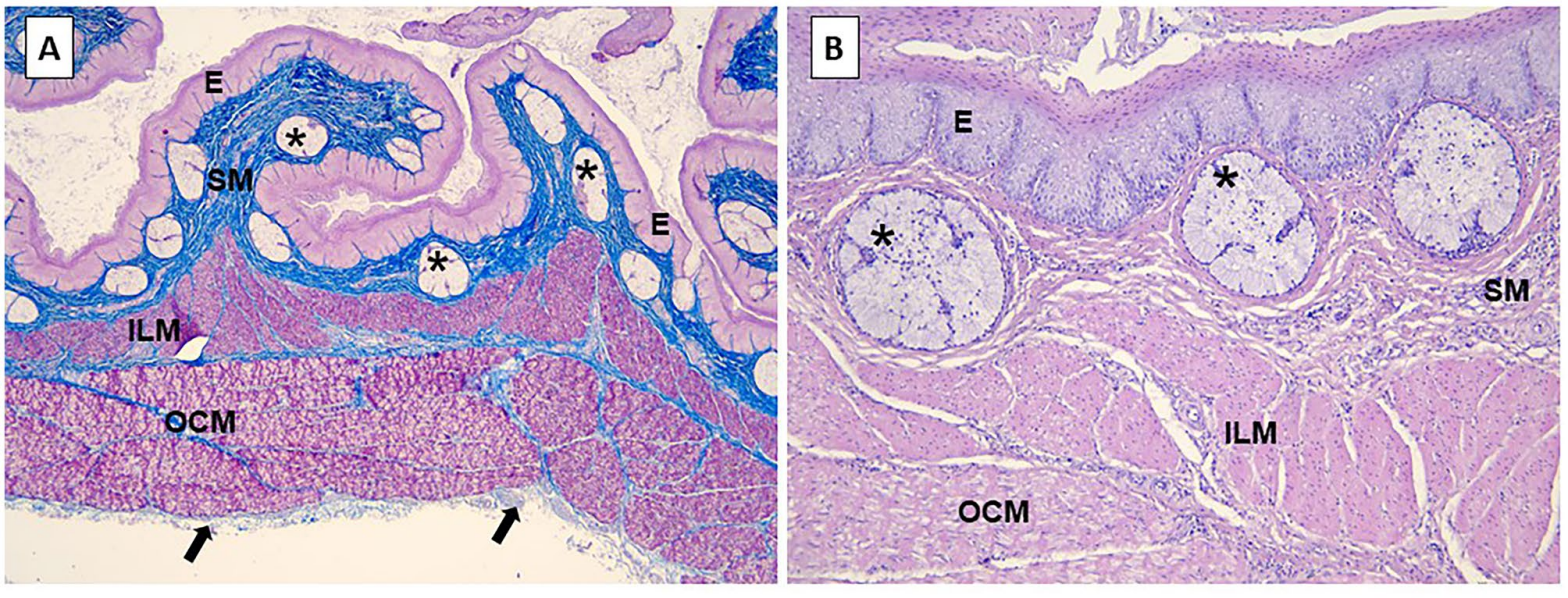
Microscopic details of the model esophagus. (**A**) Low-power view demonstrates the layered structure of the esophagus: the epithelium (E), the submucosal connective tissue (SM) with mucous glands (asterisks), inner longitudinal (ILM) and outer circumferential (OCM) layers of the muscularis propria and the adventitia (arrows); Azan stain, 25 × . (**B**) Higher magnification highlights a stratified squamous type of the epithelial lining (E), glandular structures (asterisks) within the submucosa (SM), as well as longitudinal (ILM) and circumferential (OCM) arrangement of smooth muscle bundles in the muscularis propria; H&E, 100 × .

Microscopic evaluation of duck esophagi specimens confirmed that their structure resembles that of a newborn’s esophagus (Fig. 3). Specimens used in this study contained full thickness of the mucosa, submucosal connective tissue, and muscularis propria, whereas the outermost adventitial layer was present focally. Luminal diameters of examined duck esophagi ranged from 5 to 8 mm, which is comparable to values observed in newborns and neonates^3^. Similarly, to the human counterpart, model esophagi were lined by a nonkeratinizing, stratified squamous epithelium. Beneath were folds of connective tissue with vascular structures, nerves, scattered immune cells, and mucous glands corresponding to the lamina propria mucosae/submucosa in humans. The meshwork of collagen fibers in this layer was slightly looser in areas directly adjacent to the epithelium, but there were no muscularis mucosae and no clear separation between the mucosal lamina propria and submucosa. The muscularis propria was composed of the inner, thinner, longitudinal, and outer, thicker, circumferential layer of muscle fibers. Although in humans this pattern is reversed, i.e. the outer layer is longitudinal, the relative thickness of the entire muscularis propria was similar. In a proximal portion of either human and duck esophagus, the muscular layer is formed by smooth muscles intermixed with striated muscles, but the specimens used in this experiment were taken from more distal esophageal segments and contained purely smooth muscles. The tela adventitia consisted of loose fibrous and adipose tissue with blood vessels and nerves. Overall, the degree of analogy between the esophagus of a duck and a human in relation to their histology, duck esophageal diameter matching that of a newborn as well as scrupulous preparation of the specimens preserving their key structural elements, make the applied model a good approximation of the esophagus of a young child.

### Which suture is the best for internal traction?

**Figure 4:**
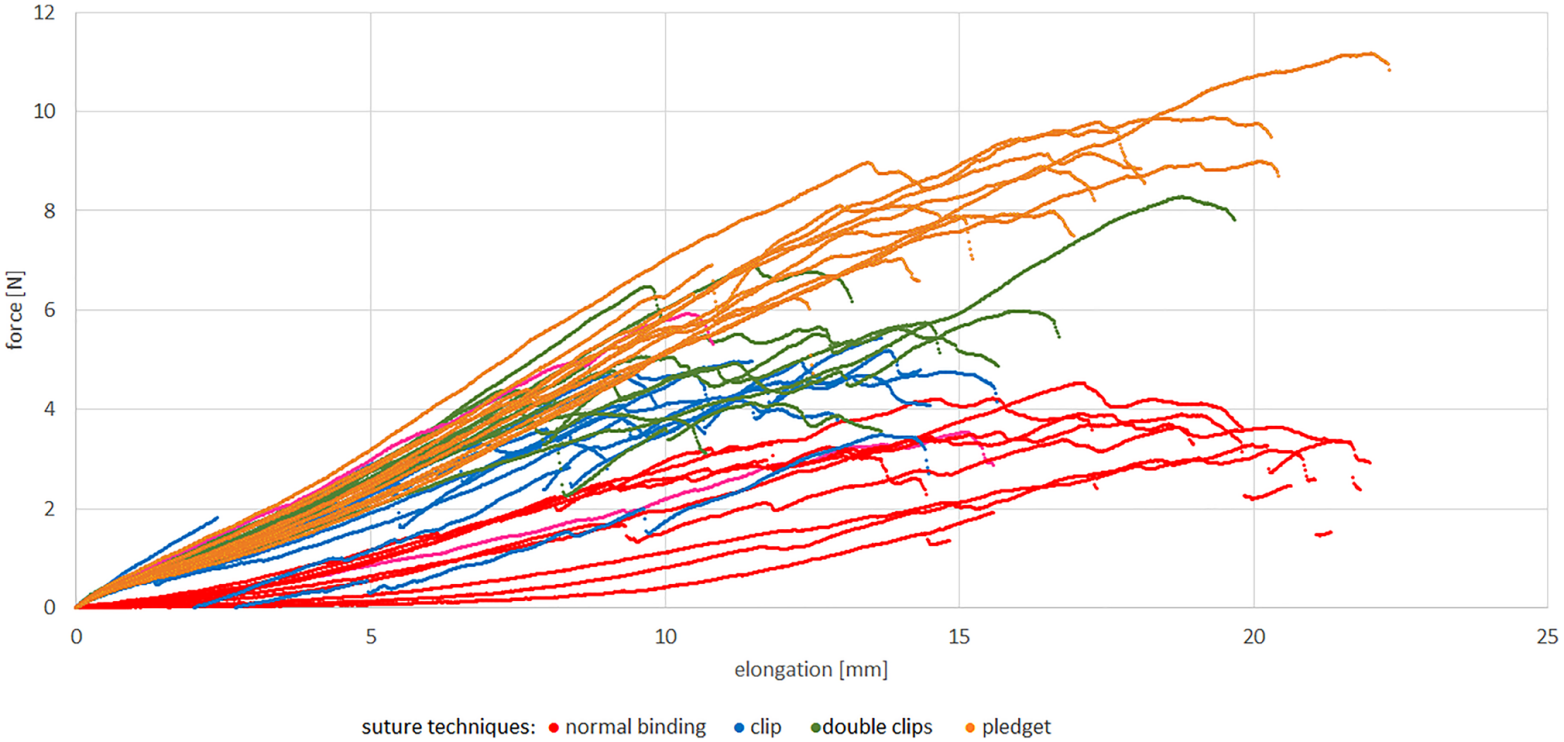
Force–elongation graphs for different sutures (normal binding means single suture; single clip—single suture reinforced with a 5 mm titan clip; double clip—single suture passed through a 5 mm double titan clip; pledget—suture with PTFE pledgets) in uniaxial traction tests.

For each sample, we created force–elongation graphs. Obtained curves are linear and have a common course for all types of sutures, but they differ in terms of biomechanical results, i.e. maximal force and tissues elongation (Fig. 4).

**Figure 5:**
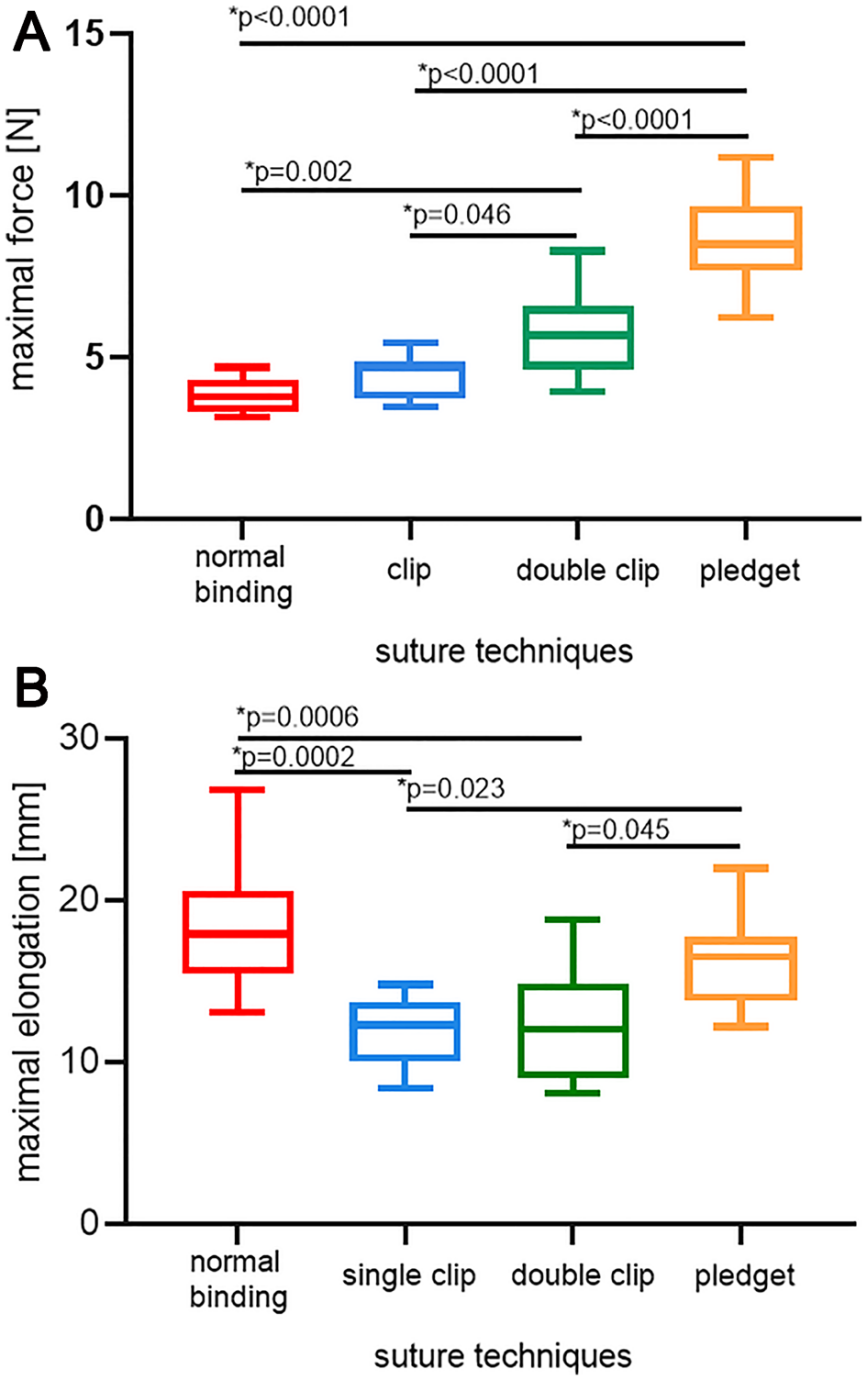
Mean maximal force (**A**) and elongation (**B**) for different types of sutures (normal binding means single suture; single clip—single suture reinforced with a 5 mm titan clip; double clip—single suture passed through a 5 mm double titan clip; pledget—suture with PTFE pledgets).

The maximal force was achieved with pledget sutures. The mean value of this parameter obtained for pledget sutures is around 57% higher in comparison with single sutures. The difference between these groups is statistically significant (*p* < 0.0001) (Fig. 5A). The maximal force achieved with clip and double clip sutures are about 48% and 33% lower comparing to the pledget sutures. These differences are statistically significant—in both cases the p value is *p* < 0.0001. The mean value of maximal force achieved with double clip is about 23% higher in comparison with the single clip. The difference is statistically significant, but the *p* value is *p* = 0.046 (Fig. 5A). The only groups that do not differ significantly in terms of maximal force are single sutures and single clips (*p* = 0.550) (Fig. 5A).

The greatest elongation was achieved with pledget sutures, and it was statistically significantly more than in single clip (*p* = 0.023) and double clip groups (*p* = 0.045) (Fig. 5B). The two latter groups (single sutures vs pledget and single clip vs double clip) did not differ significantly (*p* = 0.377 and *p* = 0.992).

### Intermittent traction gives better elongation

**Figure 6:**
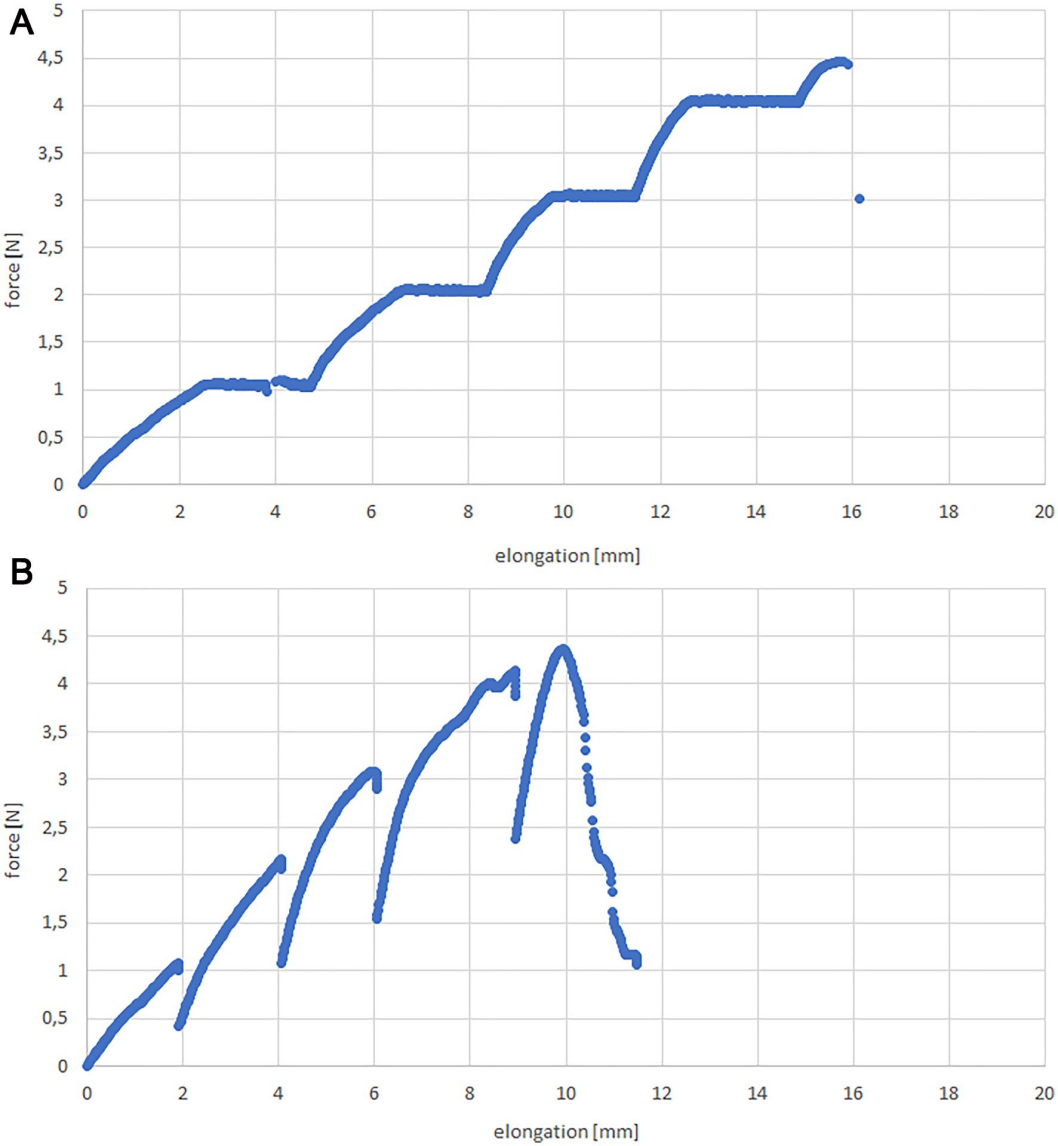
Graphs of force over elongation for simple suture with a single clip for two types of traction: (**A**) Force control, (**B**) Elongation control.

Results of intermittent traction are presented as force–elongation graphs. They have jump shaped curves different for each type of procedure (Fig. 6).

**Figure 7:**
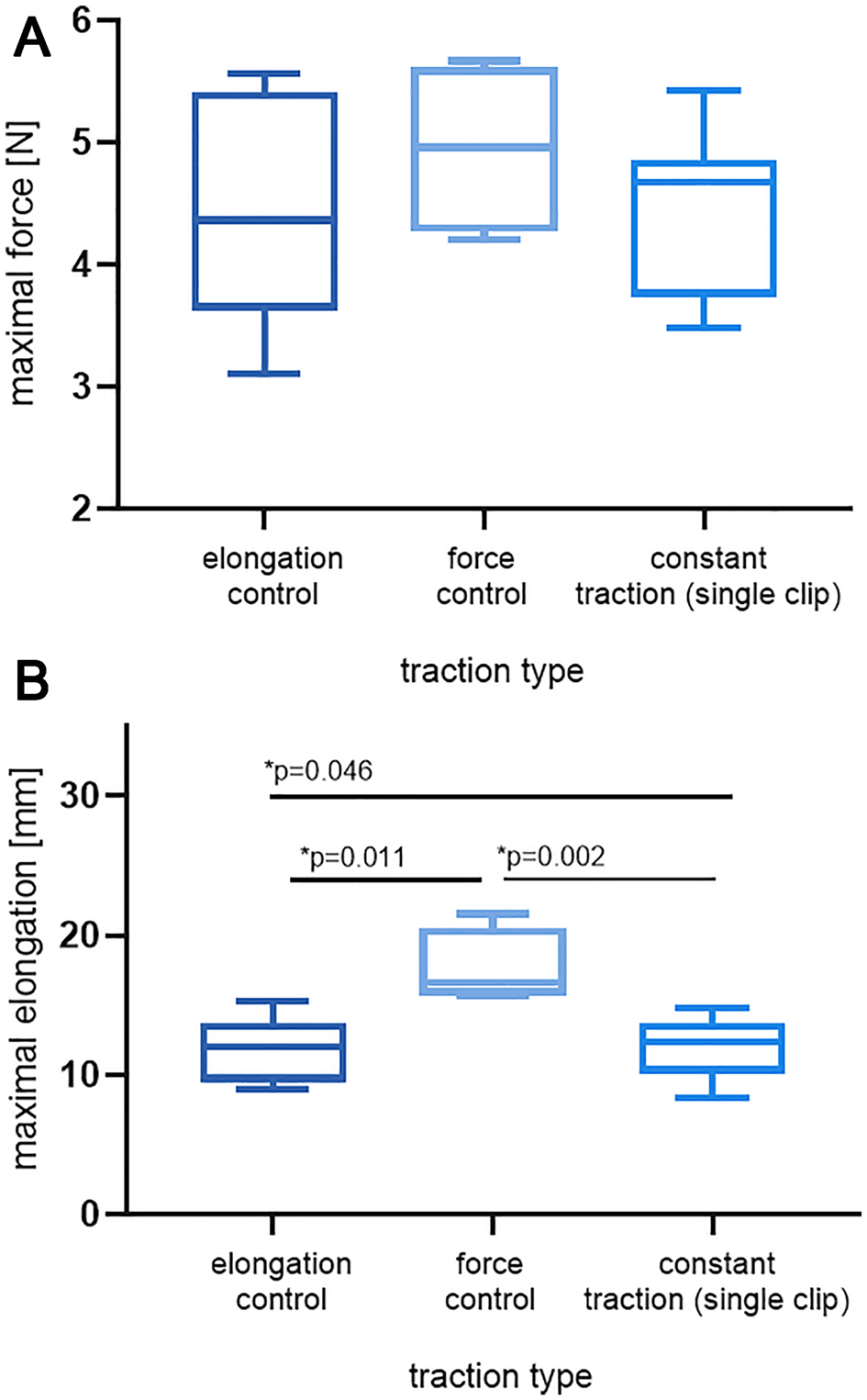
Mean maximal force (**A**) and elongation (**B**) for intermittent (Force and Elongation Control) and constant traction with single clip suture.

The mean maximal force for Force and Elongation Control was similar and the difference is non-significant (*p* = 0.459). Moreover, there is no difference between intermittent and constant traction in terms of maximal force (Elongation Control vs Force Control *p* = 0.652; Elongation Control vs Constant Traction *p* = 0.994; Force Control vs Constant Traction *p* = 0.520). Longer elongation was obtained in the Force Control group (17.59 mm vs 11.66 mm in Elongation Control; *p* = 0.011) and the result was significantly better than in Elongation Control and constant traction. Tukey’s post-hoc HSD test showed a large effect size for this test (Elongation Control vs Constant Traction *p* = 0.046) (Fig. 7).

## Discussion

### Animal model

Although swine tissues are an established animal model for biomechanical studies^4^, the goal was to best reflect the true anatomical conditions in esophageal atresia. Not only the size, shape, and texture of duck’s tissues are similar to the newborn’s esophagus, but also histological similarity, what was showed. Here it is worth noting the difference that duck esophagus lacks striated muscle cells. The distal parts of duck esophagi used in the study probably exhibits different biomechanical properties than proximal part of atretic esophagus of a newborn.

In our setting ducks’ esophagi were easy to obtain as a side product of meat production, without a need to kill animals solely for science. Duck esophagi’s small size simplifies transportation, storage and significantly decreases freezing and defrosting times. Furthermore, presented results and their normal distribution prove that this tissue choice and preparation method ensures repeatable and reliable outcomes.

### Choice of sutures

Before the selection of types of sutures for the study, we reviewed the literature on thoracoscopic esophageal traction focusing on technical aspects. Van der Zee et. al in 2007 first described thoracoscopic elongation of the esophagus using external traction^5^. They used two single sutures on each esophageal stamp and a clip at the tip of the esophagus as a radiographic marker. Another external traction was created with two purse strings on each esophageal end by Abraham^6^. The first report of internal traction appeared in 2012 when Yujiro Tanaka applied two sutures with pledgets and attached them to costal bones^7^. Similarly to van der Zee, they used a clip on the threads close to the esophagus. A subsequent technique in internal traction involved a single pledget suture also tied to a costal bone^8^.

A novel technique developed by Patkowski in our Department involves traction suture connecting both ends of the esophagus^2^. As result, the traction is applied exactly in the direction of elongation.

The simple suture was chosen as a reference point. Sutures enforced by clips had been used in our clinic for internal traction at the time we started the study, therefore they were emphasized in the study. Another question was if a double clip would excel a single clip’s advantages. Based on experiences from other centers we included pledget sutures in this study^6–8^.

The main assumption in this study is that the higher traction force we can apply without destroying the tissue, the more effective the elongation would be. Previous studies compared anastomotic sutures and constant versus intermittent traction on swine esophagi^9,10^. This is the first study to examine and directly compare different esophageal traction sutures as well as expand the knowledge on elongation and intermittent traction.

Using the single clip, we hoped to achieve better traction thanks to spreading the force over more tissues. Results show that this suture type does not lead to a higher traction force. Double clip gives a higher traction force and seals the puncture site. Results obtained with a pledget suture surpass all other types of sutures. Although this method requires double puncturing of each esophageal stump it does not seem to pose a serious problem and was successfully used in some centers. Overall results of our study testify the truth of common knowledge, that reinforced sutures (whether with a clip or a pledget), give stronger traction without damaging tissues.

Intermittent traction protocol gave better results than constant linear traction. The reason is probably tissues’ adaptation over time. The highest elongation at the same maximal force was obtained in the Force Control Group. It suggests that the constant, slowly increased tension is less damaging to the tissues than repeated periods of traction and relaxation used in the Elongation Control group. The latter probably increases microdamage and leads to earlier disruption of the esophagus.

Future studies could focus on time influence in intermittent traction as well as anastomotic sutures comparison on duck’s esophagi model. As this is preliminary experimental research on duck’s esophagi, the animal model needs further development to assess potential transferability to human.

### Limitations

The main limitation of the study is purely experimental animal model. Duck esophagus biomechanics have not been compared with human. Besides similarities in histology, there is the difference in lack of striated cells in the muscular layers. Moreover, the tissues underwent freeze–thaw cycles, which was dictated by the logistics of our project.

Another limitation is the lack of power analysis and small number of esophagi tested. Hence the present study is only of exploratory nature.

These limitations preclude direct transferability of results of our study to human but does not undermine the difference between tested sutures.

## Conclusions

Pledget and double clip should be taken into consideration in internal traction because from the biomechanical point of view they are superior to a simple suture.

When performing traction sutures, it is worth step by step carefully tightening the sliding knot in short periods before its final binding.

## Data Availability

The article contains complete data used to support the findings of this study.
